# Regulation of gene expression in the bovine blastocyst by colony stimulating factor 2

**DOI:** 10.1186/s13104-016-2038-y

**Published:** 2016-04-29

**Authors:** Manabu Ozawa, Miki Sakatani, Kyle B. Dobbs, Jasmine Kannampuzha-Francis, Peter J. Hansen

**Affiliations:** Dept. of Animal Sciences, D.H. Barron Reproductive and Perinatal Biology Research Program and Genetics Institute, University of Florida, PO Box 110910, Gainesville, FL 32611-0910 USA; Laboratory of Developmental Genetics, Institute of Medical Science, University of Tokyo, Tokyo, Japan; Kyushu-Okinawa Agricultural Research Center, National Agriculture and Food Research Organization, Kumamoto, Japan; Thermo Fisher Scientific, 5781 Van Allen Way, Carlsbad, CA 92083 USA

**Keywords:** Colony stimulating factor-2, Blastocyst, Inner cell mass, Trophectoderm, Transcriptome, Bovine

## Abstract

**Background:**

Colony stimulating factor 2 can have multiple effects on the function of the preimplantation embryo that include increased potential to develop to the blastocyst stage, reduced apoptosis, and enhanced ability of inner cell mass (ICM) to remain pluripotent after culture. The objective of the current experiment was to identify genes regulated by CSF2 in the ICM and trophectoderm (TE) of the bovine blastocyst with the goal of identifying possible molecular pathways by which CSF2 increases developmental competence for survival. Embryos were produced in vitro and cultured from Day 6 to 8 in serum-free medium containing 10 ng/ml recombinant bovine CSF2 or vehicle. Blastocysts were harvested at Day 8 and ICM separated from TE by magnetic-activated cell sorting. RNA was purified and used to prepare amplified cDNA, which was then subjected to high-throughput sequencing using the SOLiD 4.0 system. Three pools of amplified cDNA were analyzed per treatment.

**Results:**

The number of genes whose expression was regulated by CSF2, using P < 0.05 and >1.5-fold difference as cut-offs, was 945 in the ICM (242 upregulated by CSF2 and 703 downregulated) and 886 in the TE (401 upregulated by CSF2 and 485 downregulated). Only 49 genes were regulated in a similar manner by CSF2 in both cell types. The three significant annotation clusters in which genes regulated by ICM were overrepresented were related to membrane signaling. Genes downregulated by CSF2 in ICM were overrepresented in several pathways including those for ERK and AKT signaling. The only significant annotation cluster containing an overrepresentation of genes regulated by CSF2 in TE was for secreted or extracellular proteins. In addition, genes downregulated in TE were overrepresented in TGFβ and Nanog pathways.

**Conclusions:**

Differentiation of the blastocyst is such that, by Day 8 after fertilization, the ICM and TE respond differently to CSF2. Analysis of the genes regulated by CSF2 in ICM and TE are suggestive that CSF2 reinforces developmental fate and function of both cell lineages.

**Electronic supplementary material:**

The online version of this article (doi:10.1186/s13104-016-2038-y) contains supplementary material, which is available to authorized users.

## Background

Maternally-derived signals called embryokines can alter developmental phenotype of the preimplantation embryo immediately or later during pre-natal or post-natal life [1, 2]. CSF2 is one of the best-studied embryokines and has been shown to affect developmental processes in preimplantation embryo of the cow, mouse, human, and pig [2, 3]. In the cow, the species studied here, treatment of embryos with CSF2 from Day 5–7 of development can increase the proportion of embryos becoming blastocysts [4–6], block apoptosis of morula-stage embryos in response to heat shock [7], improve ability of isolated inner cell mass (ICM) to survive passage while remaining in a pluripotent state [6], increase the proportion of embryos developing to term after transfer into recipient females [8, 9] and result in calves with increased growth rate after birth [10].

The objective of the current experiment was to identify genes regulated by CSF2 in the ICM and trophectoderm (TE) of the bovine blastocyst with the goal of identifying possible molecular pathways by which CSF2 increases developmental competence for survival.

## Methods

Data were obtained as part of an experiment on gene expression in isolated ICM and TE of the bovine blastocyst that was reported earlier [11]. The earlier paper contained data from control embryos only and the current report details additional information about differences in gene expression between control embryos and embryos treated with CSF2 from Day 6 to 8 of development.

### Embryo production, isolation of ICM and TE, and RNA-Seq

These procedures were described in detail earlier [11]. Briefly, bovine embryos were produced by fertilizing slaughterhouse-derived oocytes with Percoll-purified spermatozoa from a pool of frozen-thawed semen from three bulls. Fertilization proceeded for 18–20 h and then embryos were cultured in 50 µl SOF-BE1 medium [12] at 38.5 °C in a humidified atmosphere of 5 % CO_2_ and 5 % O_2_ with the balance N_2_. At Day 6, an additional 5 µl culture medium was added containing either medium alone (control) or, for the CSF2 group, 110 ng/ml recombinant bovine CSF2 (CIBA-GEIGY, Basel, Switzerland) to achieve a final CSF2 concentration of 10 ng/ml CSF2. Blastocysts were harvested at Day 8 after fertilization and used to prepare preparations of ICM and TE using magnetic-activated cell sorting as described [11]. Three separate pools of TE and ICM for each treatment were obtained. Each pool was prepared using 88–102 blastocysts.

Total RNA isolated from each pool of embryonic cells was used to prepare amplified cDNA using the Ovation RNA-Seq kit (NuGen Technology, San Carlos, CA, USA). Barcoded fragment libraries were constructed using the SOLiD™ v4 fragment library kit. Amplified bead-bound libraries were clonally amplified by emulsion PCR according to the Applied Biosystems SOLiD™ 4 Systems Templated Bead Preparation Guide (Applied Biosystems, Foster City, CA, USA). Clonally amplified template DNA were P2-enriched and extended with a bead linker by terminal transferase. Approximately 600–700 M beads were deposited on each slide and sequenced using ‘sequencing by ligation’ chemistry and the 50 × 5 bp protocol on the SOLiD™ v4 sequencer (Applied Biosystems) at the Interdisciplinary Center for Biotechnology Research, University of Florida. Results were obtained as color space fasta files. Processing of reads was described earlier [11]. A total of 64.4 % or 595 million reads were mapped successfully. Data were deposited as Submission DRA000504 in the DDBJ Sequence Read Archive at http://www.ddbj.nig.ac.jp/index-e.html.

### Analysis of RNA-Seq data

The number of mapped reads for each individual gene was counted using the HTSeq tool (http://www-huber.embl.de/users/anders/HTSeq/doc/overview.html) with intersection-nonempty mode. The DESeq package [13] in R was used to determine treatment effects on gene expression. Treatment effects were determined by making the following comparisons: ICM-control vs ICM-CSF2 and TE-control vs TE-CSF2. Two separate sets of differentially expressed genes were identified. The first included those with a P value of <0.05 and a minimum fold-change of >1.5 or <0.66 (CSF2/control) and the second included the same fold-change cut-off and a P < 0.05 after adjustment for a 2 % false discovery rate using the Benjamini-Hochberg procedure [14].

### Bioinformatics

The differentially expressed genes in TE and ICM were analyzed by the Database for Annotation, Visualization and Integrated Discovery (DAVID; (DAVID Bioinformatics Resources 6.7, http://david.abcc.ncifcrf.gov/) [15]. Ambiguous gene identifiers were submitted to the gene conversion tool to create DAVID gene names. The DAVID database was queried to identify functional annotation terms and clusters in which differentially expressed genes were overrepresented. Clusters of annotation terms with Benjamini-Hochberg corrected P values of <0.05 were identified. Lists of upregulated and downregulated genes in ICM and TE were also separately analyzed for overrepresentation of genes in SuperPaths using GeneAnalytics (https://ga.genecards.org). SuperPaths are a collection of 1073 unified pathways generated from multiple pathway sources using nearest neighbor graphing and hierarchical clustering [16]. All SuperPaths with an adjusted P value of <0.0001 are reported as well as selected paths with an adjusted P value of <0.05. Lists of differentially expressed genes in ICM and TE were separately uploaded to Ingenuity Pathway Analysis (IPA, Qiagen, Redwood City, CA, USA) for 1) clustering of differentially expressed genes into diseases and functions predicted to be changed as a result of differential gene expression and 2) identification of putative transcriptional regulators of differentially expressed genes. A function was predicted to be changed when the activation z-score ≥2.0. For upstream regulators, transcriptional regulators that were predicted to be activated or inhibited by CSF2 (i.e., activation z-score ≥2.0) are reported.

## Results

### Genes regulated by CSF2

The effects of CSF2 on gene expression are described in Additional file 1. The number of genes whose expression was regulated by CSF2, using P < 0.05 and >1.5-fold or <0.66-fold difference as cut-offs, was 945 in the ICM (242 upregulated by CSF2 and 703 downregulated) and 886 in the TE (401 upregulated by CSF2 and 485 downregulated). A total of 74 genes were regulated by CSF2 in both ICM and TE (Additional file 1). Of these, 9 were upregulated by CSF2 in both cell types, 40 were downregulated by CSF2 in both cell types, 13 were upregulated by CSF2 in ICM and downregulated in TE and 12 were downregulated in ICM and upregulated in TE.

When a more stringent criterion was applied, an adjusted P value of <0.05 after performing a Benjamini-Hochberg correction for a false discovery rate of 2 %, CSF2 changed expression of 25 genes in the ICM (8 upregulated and 17 downregulated by CSF2) and 23 genes in the TE (14 upregulated and 9 downregulated). The list of these genes for ICM is shown in Table 1. The largest increase in expression caused by CSF2 was for *FRMPD3* (14.7-fold), *NDNF* (13.3-fold), *LAPTM5* (12.3-fold) and an ortholog of *OLFR1239* (8.5-fold). The largest decrease in expression was for a bovine ortholog of *MYADM* (undetectable in cells from CSF2-treated embryos), olfactory receptor, family 5, subfamily T like (0.11control/CSF2 or a 19.9-fold decrease), *CMYA5* (19.7-fold decrease) and another family 5 olfactory receptor (*OR5T2;* 8.8-fold decrease).

**Table 1:** Genes regulated by CSF2 in inner cell mass after adjustment for a false discovery rate of 2 %

Gene symbol or description	Mean counts, control	Mean counts, CSF2	Fold change, CSF2/control	Adjusted P value
Upregulated				
*FRMPD3*	1.4	21.3	14.71	0.045
*NDNF*	1.7	22.8	13.29	0.019
*LAPTM5*	43.3	531.8	12.28	0.000
Olfactory receptor 4A47 (LOC100294956)	4.4	37.2	8.48	0.014
*CYP3A5*	37.3	230.9	6.20	0.000
Serine incorporator 1 pseudogene (LOC781002)	25.2	152.2	6.05	0.000
*TMEM182*	17.8	83.6	4.69	0.014
*CSN3*	97.6	444.2	4.55	0.000
Downregulated				
*MYADM*-like (ENSBTAG00000001526)	10.2	0	–	0.006
*CD68*	1156.8	491.9	0.43	0.024
*CFAP54*	162.4	69.2	0.43	0.026
*DAG1*	345.6	141.4	0.41	0.026
*HSD3B1*	183.6	71.7	0.39	0.002
EKC/KEOPS complex subunit LAGE3 (LOC618023)	87.4	29.9	0.34	0.019
*SEMA4G*	127.1	42.5	0.33	0.026
*BAG3*	835.4	204.9	0.25	0.028
*PAQR7*	187.9	31.5	0.17	0.013
*PAH*	56.2	8.8	0.16	0.012
*MUC3A*	79.6	12.4	0.16	0.014
*SOX30*	85.3	12.6	0.15	0.013
*HSPA6*	240.0	29.3	0.12	0.000
*SYNE2*	635.1	76.7	0.12	0.000
*OR5T2*	88.6	10.1	0.11	0.000
Olfactory receptor, family 5, subfamily T-like (ENSBTAG00000039348)	11.7	0.6	0.05	0.046
*CMYA5*	38.5	2.0	0.05	0.006

Results for genes regulated by CSF2 in TE after correcting for multiple testing are summarized in Table 2. The largest increase in expression caused by CSF2 was for *CLIC6* (undetectable in the absence of CSF2 and present in CSF2-treated TE), the olfactory receptor *OR5AN1* (16.2-fold), *CSN3* (13.1-fold; also upregulated 4.6-fold in ICM) and a uncharacterized gene (11.5-fold). The largest decrease was for a gene orthologous to interferon-regulatory factor (19.2-fold decrease), *NPY1R* (17.8-fold decrease), *PAQR7* (17.0-fold decrease) and *VAV3* (4.8-fold decrease).

**Table 2:** Genes regulated by CSF2 in trophectoderm after adjustment for a false discovery rate of 2 %

Gene symbol or description	Mean counts, control	Mean counts, CSF2	Fold change, CSF2/control	Adjusted P value
Upregulated				
*CLIC6*	0	6.7	–	0.025
*OR5AN1*	0.6	9.7	16.22	0.021
*CSN3*	42.4	554.8	13.08	0.000
Uncharacterized (ENSBTAG00000033304)	11.1	127.5	11.46	0.000
Exocyst complex component 1-like (LOC100335745)	1.2	9.9	8.09	0.049
Uncharacterized (ENSBTAG00000040552)	2.9	20.8	7.19	0.012
*HPCAL4*	427.9	2667.1	6.23	0.000
*CCDC146*	12.9	70.3	5.43	0.000
DEAD/H box polypeptide 26B-like (LOC100138355)	7.4	34.9	4.72	0.002
Uncharacterized (LOC100335677)	17.2	68.7	3.99	0.012
*TKTL1*	64.6	205.7	3.18	0.001
*FOS*	344.1	775.3	2.25	0.042
*DUSP1*	897.1	1959.3	2.18	0.008
*SHE*	216.5	445.6	2.06	0.018
Downregulated				
*SLC25A40*-like (LOC510700)	451.6	179.4	0.40	0.045
*AKAP4*	104.7	30.0	0.29	0.004
*MUC1*	284.2	73.8	0.26	0.000
*VAV3*	43.1	10.3	0.24	0.003
*VAV3*	52.6	11.5	0.22	0.002
*VAV3*	34.1	7.0	0.21	0.001
*PAQR7*	341.6	20.1	0.06	0.000
*NPY1R*	333.9	18.8	0.06	0.000
Interferon regulatory factor-like (ENSBTAG00000031214)	26.4	1.4	0.05	0.000

### Functional annotation of differentially expressed genes

A total of 671 differentially expressed genes in the ICM was annotated by DAVID. There were three annotation clusters having Benjamini-Hochberg adjusted P values <0.05 (Additional file 2). These included a cluster of 194 genes related to membrane signaling (enrichment score = 4.26), another of 254 genes related to membrane and extracellular space (enrichment score = 2.94) and a cluster of 30 genes related to sensory perception (enrichment score = 2.36). Of note was the large number of differentially expressed genes associated with G-protein coupled receptor protein signaling pathway (67 genes) and olfactory receptor activity (48 genes, adjusted P value = 0.013). CSF2 increased expression of some olfactory genes and decreased expression of others. For example, *LOC100294956* (olfactory receptor 4A47) was increased 8.48-fold and *OR5T2* was decreased 9.09-fold (Table 1). The absolute number of reads for most putative olfactory receptor genes was low (<5).

For differentially expressed genes in the TE, 648 differentially expressed genes were annotated by DAVID. The only cluster of pathways with a Benjamini-Hochberg adjusted P value <0.05 was for 108 genes related to secreted or extracellular proteins (enrichment score = 4.22) (Additional file 2).

GeneAnalytics was used to identify SuperPaths in which genes regulated by CSF2 were overrepresented. Using a P value of 0.001 as a cutoff, there were no SuperPaths identified for genes upregulated by CSF2 in ICM. Among genes downregulated by CSF2 were those in the ERK signaling, integrin pathway, visual cycle and AKT signaling (Table 3). There were 11 Superpaths in which genes upregulated by CSF2 in TE were overrepresented (Table 4). Among these were those for apoptotic pathways in synovial fibroblasts, G protein signaling Ras family GTPases in kinase cascades, and ERK signaling. There were four SuperPaths containing genes downregulated by CSF2 in TE with P < 0.001 including for CREB pathway, actin nucleation by ARP-WASP complex, Akt signaling, and ERK signaling (Table 5). In addition, there were two SuperPaths related to pluripotency and differentiation of the blastocyst (TGF-β, Nanog in mammalian ESC pluripotency) that contained an overrepresentation of downregulated genes in the TE at P < 0.05.

**Table 3:** SuperPaths overrepresented among genes downregulated in the inner cell mass by CSF2

ERK signaling (score = 22.99)—51 genes
*ACTA2, ACTG2, ADCY8, ALK, ARHGDIB, ARHGEF6, CACNB3, CACNA1G, CCL5, CDH2, CDH9, COL1A1, DES, EFEMP1, EPHA3, ESR2, FGF16, FOSB, FOSL1, GNA11, GNA12, GNAT2, IL11RA, IL12RB2, IL15RA, IL20, IL4, IL7, IRAK1, LEF1, MAPK12, MYH1, MYLPF, MYO18B, MYO7A, MUC1, MYC, NCAN, PAK3, PPP1R3C, SEMA4G, SMAD9, SOX11, SOX30, STAT6, TGFBR3, TIMP2, TNFAIP6, TNFSF9, TNFSF11, VIM*
Integrin pathway (score = 16.42)—28 genes
*ACTA2, ACTG2, ADCY8, ARHGEF6, CACNA1G, CACNB3, CAV1, CCL5, CLDN1, COL1A1, EFEMP1, GNA11, GNA12, GNAT2, MAPK13, MMP9, MYC, MYH1, MYLPF, MYO18B, MYO7A, NCAN, NOX1, PAK3, STAT6, TGFBR3, THBS1, VAV3*
Visual cycle (score = 14.74)—10 genes
*GNA11, GNA12, GNAT2, GUCA1A, GUCY2C, LRAT, PDE6A, RDH10, RDH12, SLC24A1*
Akt signaling (score = 14.45)—30 genes
*ACTA2, ACTG2, AKR1B1, ALK, ATXN7, CCL5, EPHA3, FGF16, FOSL1, GABRG1, GNA11, GNA12, GNAT2, IL11RA, IL12RB2, IL15RA, IL20, IL4, IL7, IRAK1, MAPK13, PAK3, SEMA4G, STAT6, TIMP2, TLR7, TNFAIP6, TNFSF11, TNFSF9, VAV3*

Shown are those SuperPaths that were highly significant (P < 0.001). Note that there were no SuperPaths identified in the list of genes upregulated in the ICM by CSF2

**Table 4:** SuperPaths overrepresented among genes upregulated in the trophectoderm by CSF2

Inhibitory actions of lipoxins on superoxide production in neutrophils (score = 25.39)—9 genes
*FOS, GNG12, GNG2, GNGT2, JUN, PLD1, NFKBIA, PIK3CG, PREX1*
Translation regulation by alpha-1 adrenergic receptors (score = 19.66)—8 genes
*ADRA1A, FOS, GNG12, GNG2, GNGT2, JUN, PLD1, PRKCE*
Hypertrophy model (score = 18.66)—5 genes
*ANKRD1, ATF3, CYR61, HBEGF, IFNG*
Apoptotic pathways in synovial fibroblasts (score = 16.29)—22 genes
*ANK1, ARHGDIB, CACNA2D1, CTGF, FOS, GNG12, GNG2, GNGT2, HBEGF, ITGB3, JUN, KITLG, KLF10, MAP3K8, NFKBIA, NRP1, PLD1, PPARA, PPP1R14A, PRKCE, RHOB, ZAP70*
G-protein signaling ras family gtpases in kinase cascades (score = 15.90) 8 genes
*CYR61, FOS, FOSB, JUN, LDLR, NFKBIA, PRKCE, SIPA1*
Toll comparative pathway (score = 15.54) 8 genes
*ATF3, FOS, FOSB, GNG2, IFNG, IL1RAP, JUN, NFKBIA*
ERK signaling (score = 15.12) 29 genes
*ARHGDIB, ATF3, CACNA2D1, CAPN11, CTGF, DUSP1, FOS, FOSB, GNG12, GNG2, GNGT2, HBEGF, IL17A, ITGB3, JUN, KITLG, KLF10, MAP3K8, NFKBIA, NRP1, PIK3CG, PLD1, PPP1R14A, PRKCE, RHOB, SMAD9, SPP1, TNFRSF8, ZAP70*
Osteopontin-mediated events (score = 15.0)—5 genes
*FOS, ITGB3, JUN, NFKBIA, SPP1*
ATF-2 transcription factor network (score = 14.65)—6 genes
*ATF3, DUSP1, DUSP5, FOS, IFNG, JUN*
Angiotensin activation of ERK (score = 14.51)—11 genes
*FOS, GNG12, GNG2, GNGT2, HBEGF, KITLG, ITGB3, JUN, PIK3CG, PRKCE, ZAP70*
LPA receptor mediated events (score = 13.79)—6 genes
*FOS, GNG2, HBEGF, JUN, NFKBIA, PRKCE*

Shown are those SuperPaths that were highly significant (P < 0.001)

**Table 5:** SuperPaths overrepresented among genes downregulated in the trophectoderm by CSF2

CREB Pathway (score = 17.43)—22 genes
*ARHGEF6, AXL, BMP3, CACNA2D3, CACNG6, CLU, EPHA3, EPHB3, FLT1, FLT3, GNA14, GNB5, GNRH1, GRID2, HTR3E, PDE1C, PRKCH, SEMA5A, SLC8A2, TEK, THPO, VAV3*
Actin Nucleation by ARP-WASP Complex (score = 14.94)—16 genes
*ARHGEF6, AXL, EPHA3, EPHB3, FLT1, FLT3, GNA14, GNB5, ITGAE, ITGB6, LPAR1, LPAR3, MYH1, MYH7, MYO7A, TEK*
Akt Signaling (score = 14.53)—24 genes
*AXL, BMP15, BMP3, CCL17, CD28, CD80, EPHA3, EPHB3, FCER1G, FLT1, FLT3, GNA14, GNB5, GNRH1, IL10, ITGAE, ITGB6, PRKCH, PXDN, SEMA5A, TEK, THPO, TNFSF8, VAV3*
ERK Signaling (score = 14.11)—34 genes
*ARHGEF6, AXL, BMP15, BMP3, CACNA2D3, CACNG6, CCL17, CD28, CD80, CDH13, ECM2, EPHA3, EPHB3, FBN1, FLT1, FLT3, GNA14, GNB5, GNRH1, IL10, ITGAE, ITGB6, LAMA4, MUC1, MYC, MYH1, MYH7, MYO7A, PRKCH, PXDN, SEMA5A, TEK, THPO, TNFSF8*
TGF-BETA (score = 11.30) 21 genes
*ARHGEF6, AXL, BMP15, BMP3, CCL17, CD80, EPHA3, EPHB3, FLT1, FLT3, GNA14, GNB5, GNRH1, IL10, ITGAE, ITGB6, MYC, PXDN, SEMA5A, TEK, THPO*
Nanog in mammalian ESC pluripotency (score = 9.82)—17 genes
*AQP2, AXL, BMP3, CDH13, EPHA3, EPHB3, FLT1, FLT3, GNB5, GNRH1, LPAR1, LPAR3, PRKCH, SEMA5A, TEK, THPO, WNT8B*

Shown are those SuperPaths that were highly significant (P < 0.001) as well as two selected pathways that were P < 0.05 (TGF-BETA and Nanog in Mammalian ESC pluripotency)

Sets of genes that were differentially expressed in ICM and TE were separately subjected to analysis by IPA to determine 1) diseases and functions predicted to be activated or inhibited as a result of changes in gene expression caused by CSF2 and 2) transcriptional regulators that were postulated to be activated or inhibited in response to CSF2. For ICM, a total of 22 functions (many overlapping) were predicted to change in response to alterations in gene expression caused by CSF2 (Additional file 3). In all cases, the function was predicted to decrease. Among these functions were 9 related to phagocytosis or cell movement, 2 related to changes in cell morphology, and 3 related to immune responses. For TE, there were 6 functions predicted to change in response to CSF2 and all were predicted to decrease. These functions were related to antibody response, quantity of cells and cell migration, and dermatitis. Three transcription factors were predicted to be activated by CSF2 in ICM (HLX, IKZF1, and ZNF217) while 13 were predicted to be inhibited (HSF1, STAT3, SRF, STAT4, CREM, SP1, CREB1, FOXM1, ERG, ETS1, SMARCA4, CREBBP and CTNNB1) (Additional file 3). Only two transcription factors were predicted to be activated by CSF2 in TE (SATB1 and NFATC2) and none were predicted to be inhibited.

### Comparison to genes regulated by CSF2 in bovine morulae and mouse blastocysts

The set of genes regulated by CSF2 in the ICM and TE were compared to sets of genes previously reported to be regulated by CSF2 in embryos. In an earlier experiment with bovine embryos, Loureiro el al. [7] identified 67 genes in Day 6 morula that were upregulated by CSF2 treatment at Day 5 and 92 genes that were downregulated. Of these 159 genes, only 7 were regulated by CSF2 in a similar manner for Day 8 blastocysts. Of genes upregulated by CSF2 in morula, *SLC26A5* was upregulated in ICM, *ANKRD37* was upregulated in TE, and *PYGO1* was upregulated in ICM and TE. Of genes downregulated in morula, *DTX3* and *VGLL1* were downregulated in ICM and *NEDD4* and *PHGDH* were downregulated in TE.

In another experiment, Chin et al. [17] identified 16 genes related to stress responses that were downregulated in mouse blastocysts treated with CSF2. Two of these genes, *DFF5* and *HSPH1*, were also downregulated in ICM in the current experiment.

## Discussion

CSF2 can have multiple effects on the function of the developing bovine embryo. These include increased potential to develop to the blastocyst stage [4–6], reduced apoptosis [7], and enhanced ability of ICM to remain pluripotent after culture [6]. As a result of these or other actions, embryos treated with CSF2 have increased ability to develop to term after transfer to recipients [8, 9]. Accordingly, CSF2 could be a useful molecule for commercial production of bovine embryos based on in vitro fertilization and culture. In the current study, it was shown that effects of CSF2 on embryonic function are accompanied by changes in gene expression in both the ICM and TE of the blastocyst. Moreover, the actions of CSF2 on gene expression were largely distinct for the two cell types. Of 1782 genes regulated by CSF2 > 1.5-fold, only 49 genes were regulated by CSF2 in the same direction in both cell types. Thus, differentiation of the blastocyst is such that, by Day 8 after fertilization, the ICM and TE respond differently to maternal signals. Analysis of the genes regulated by CSF2 in ICM and TE are suggestive that CSF2 reinforces developmental fate and function of both cell lineages. Actions of CSF2 on the ICM reduced expression of genes in signaling pathways involved in differentiation while actions of CSF2 on the TE reduced expression of genes in pluripotency pathways and may have increased capacity of the TE to interact with the maternal system through upregulation of genes involved in extracellular processes.

Many of the genes regulated by CSF2 in the ICM are involved in signal transduction. The three significant annotation clusters in which genes regulated by CSF2 were overrepresented are related to membrane signaling. The overall picture is for downregulation of genes involved in signal transduction, particularly for the ERK/MAPK and AKT signaling pathways. Repression of MAPK signaling by CSF2 may facilitate maintenance of the ICM in a pluripotent condition. In cattle, inhibition of MAPK signaling increases numbers of cells positive for NANOG while reducing expression of the TE gene *IFNT* and cells positive for the hypoblast marker GATA6 [18, 19]. Inhibition of MAPK also promotes formation of naïve-type induced pluripotent stem cells in the cow [20]. Downregulation of genes involved in AKT signaling would counteract the downregulation of MAPK signaling because this pathway can itself inhibit MAPK signaling [21]. However, inhibition of PI3K (upstream regulator of AKT) did not affect expression of pluripotency-related genes in the bovine blastocyst although ICM numbers were reduced [22]. CSF2 can signal through the phosphatidylinositol 3-kinase/AKT pathway [23] and downregulation of expression of genes in the AKT pathway by CSF2 could reflect feedback inhibition. Three transcription factors that are involved in actions of CSF2 in other cells, CTTNB1, SP1, and STAT3 [24, 25], were also predicted to be inhibited in the ICM by CSF2.

Another indication that CSF2 promotes maintenance of the ICM in a pluripotent state was the observation that the transcription factor ETS1 was predicted to be inhibited in the ICM. ETS1 promotes differentiation of human trophoblast [26] and a related molecule, ETS2, is involved in transcriptional regulation of trophoblast specific genes like *IFNT* and *TKDP1* [27]. Another transcriptional regulator predicted to be inhibited by CSF2 in the ICM, STAT3, is important for formation of the ICM and expression of pluripotency markers in cattle [22] while another, CREBBP participates in downregulation of the trophoblast gene *IFNT* [28].

The TE is the cell type in the blastocyst that interacts most closely with the maternal reproductive tract, both because of its location at the outer boundaries of the embryo and because tight junctions between TE cells [29] limit movement of molecules from the ICM to the extra-embryonic space. CSF2 may facilitate interactions between the TE and maternal cells because genes regulated by CSF2 in the TE included a large cluster of genes related to secreted or extracellular proteins. Among the genes whose expression in TE was downregulated by CSF2 was *MUC1*, which encodes for a mucin that inhibits adhesion to cell surfaces. MUC1 is present on human trophoblast and overexpression in the Jar trophoblast cell line reduced invasiveness of the cells [30]. Perhaps inhibition of *MUC1* expression by CSF2 increases the competence of the TE to interact with the endometrium. In addition, MUC1 can participate in cell signaling because its cytoplasmic tail can regulate β-catenin stability [31].

One gene whose expression in TE was increased by CSF2 was *FOS.* The promoter for the maternal recognition of pregnancy protein, *IFNT*, contains an AP1 site [32] and deletions in that site can decrease *IFNT1* gene expression [27]. Treatment of embryos with CSF2 from Day 5–7 caused an increase in IFNT accumulation in the uterus at Day 15 of pregnancy for male embryos [33] and it is possible that regulation of *FOS* is involved in this action of CSF2. If so, effects of CSF2 could be sex-specific because CSF2 decreased IFNT accumulation in the uterus of cows that received female embryos [33].

CSF2 may also promote differentiation of the TE. Unlike for the mouse, the TE of the bovine embryo only gradually becomes committed to the TE fate and, when aggregated with 8-cell embryos, some TE cells from the day 7 blastocyst can revert to ICM [34]. Several genes downregulated by CSF2 in TE were identified as members of the Nanog and TGF-β signaling pathways. NANOG is a well-described promoter of pluripotency in the ICM [35] and TGF-β signaling has been reported to promote pluripotency in human embryonic stem cells [36] but to reduce epiblast formation in the mouse embryo [37].

One of the surprising set of signaling genes regulated by CSF2 in the ICM was that for olfactory receptors. Some of these genes were increased in expression by CSF2 and others decreased. Several olfactory receptors were also regulated by CSF2 in the TE including *OR5AN1*, which was increased 16-fold by CSF2 (Table 2). Olfactory receptors are a large gene family. A total of 1071 olfactory receptor related sequences were identified in the bovine genome including 881 functional genes with the remainder being pseudogenes or partial sequences [38]. The fact that specific olfactory receptors are regulated by CSF2 implies that olfactory receptor signaling may be important for embryonic development and that actions of specific odorants on the embryo could be modified by CSF2.

CSF2 is best known for its role in the immune system, where in addition to promoting formation of granulocytes and macrophages from hematopoietic stem cells, it also controls neutrophil function and macrophage differentiation [39, 40]. Regulation of genes involved in immune function by CSF2 in the embryo accounts for the observation that many of the functions predicted to change in response to CSF2 were related to immune function.

The finding that the genes regulated by CSF2 in the ICM were distinct from those regulated by CSF2 in the TE strengthens the idea that transcriptional regulation by CSF2 depends on cell type. In addition, the genes regulated by CSF2 in the ICM and TE were largely different than those found to be regulated by CSF2 in the bovine morula [7]. The 7 genes that were regulated similarly by CSF2 in morula and blastocysts include 3 upregulated genes (*ANKRD37,* a putative nuclear localization signal, *PYGO1*, a coactivator of transcription of WNT-regulated genes, and *SLC26A5,* an incomplete anion transporter involved in changing cell shape) and 4 downregulated genes (*DTX3* and *NEDD4,* both involved in ubiquitination, *PHGDH,* an enzyme involved in serine synthesis, and *VGLL1,* a coactivator of transcription factors). In the bovine, activation of WNT signaling either reduced [41, 42] or increased [41] competence of embryos to become blastocysts and preferentially reduced number of TE cells as compared to ICM cells [42]. Deubiquitinating enzymes are required for blastocyst formation in mice and pigs [43]. Serine is probably not a major energy source for the bovine embryo because it is not taken up by blastocysts to a large degree [44] and addition of the amino acid to culture medium of bovine embryos had no effect on development to the blastocyst stage [45].

## Conclusions

CSF2 can cause differential actions on gene expression in the ICM and TE. One interpretation of changes in gene expression is that CSF2 reinforces the lineage commitment decisions in ICM and TE and enhances ability of the TE to communicate with the maternal reproductive tract. Additional functional studies will be required to test these hypotheses.

## Additional files

10.1186/s13104-016-2038-y Gene expression in ICM and TE as affected by CSF2. Tab 1: Gene expression in the ICM. Tab 2: Genes whose expression in the ICM was affected by CSF2 (P < 0.05; >1.5 or <0.66 fold change). Note that genes highlighted in red (upregulated) or green (downregulated) were significantly upregulated by CSF2 after correcting for a 2 % false-discovery rate Tab 3: Gene expression in the TE. Tab 4: Genes whose expression in the TE was affected by CSF2 (P < 0.05; >1.5 or <0.66 fold change). Coloring code is as for Tab 3. Tab 5: Genes that were regulated by CSF2 in both the ICM and TE.
10.1186/s13104-016-2038-y Annotation clusters of gene ontologies identified by DAVID for genes regulated by CSF2 in ICM (Tab 1) and TE (Tab 2). Only clusters having Benjamini-Hochberg adjusted P values <0.05 are shown.
10.1186/s13104-016-2038-y Analysis of genes regulated by CSF2 using IPA. Shown are diseases or functions predicted to be increased or decreased as a result of CSF2 treatment for ICM (Tab 1) and TE (Tab 2) as well as transcriptional regulators functions predicted to be activated or inhibited by CSF2 in ICM (Tab 3) and TE (Tab 4). For all tabs, rows are highlighted (red, activated; green, inhibited) when the activation z-score ≥ 2.0.

## Abbreviations

ICM: inner cell mass
TE: trophectoderm
